# Modulation of the Intestinal Microbiota by the Early Intervention with Clostridium Butyricum in Muscovy Ducks

**DOI:** 10.3390/antibiotics10070826

**Published:** 2021-07-07

**Authors:** Xingning Xiao, Zixian Fu, Na Li, Hua Yang, Wen Wang, Wentao Lyu

**Affiliations:** 1 State Key Laboratory for Managing Biotic and Chemical Threats to the Quality and Safety of Agro-Products, Institute of Agro-Product Safety and Nutrition, Zhejiang Academy of Agricultural Sciences, Hangzhou 310021, China; xingningxiao@126.com (X.X.); iyyf2765036567@163.com (Z.F.); 2018820662006@stu.zafu.edu.cn (N.L.); yanghua@mail.zaas.ac.cn (H.Y.); wangwen@zaas.ac.cn (W.W.); 2 College of Animal Science, Zhejiang A&F University, Hangzhou 310058, China

**Keywords:** *Clostridium butyricum*, Muscovy ducks, intestinal microbiota, short-chain fatty acids

## Abstract

This study evaluated the effects of early intervention with *Clostridium butyricum* (*C. butyricum*) on shaping the intestinal microbiota of Muscovy ducklings. A total of 160 1-day-old male ducks were randomly divided into two groups: the CB group was administered with 1 mL of *C. butyricum* (2 × 10^9^ CFU/mL), while the C group was given 1 mL of saline. The administration lasted for 3 days. We found that *C. butyricum* had no significant effect on growth performance. The results indicated that inoculation with *C. butyricum* could significantly increase the abundance of genera *Bacteroides*, *Lachnospiraceae*_uncultured, and *Ruminococcaceae* on Day 14 and reduce the abundance of *Escherichia–Shigella* and *Klebsiella* on Days 1 and 3. Moreover, the CB group ducks had higher concentrations of acetic, propionic, and butyrate in the cecum than the C group. Overall, these results suggest that early intervention with *C. butyricum* could have positive effects on Muscovy ducks’ intestinal health, which might be attributed to the modulation in the intestinal microbial composition and the increased concentrations of short-chain fatty acids (SCFAs). *C. butyricum* might even have the potential to help the colonization of beneficial bacteria in the intestine microbiota in Muscovy ducks in poultry and other livestock.

## 1. Introduction

In recent years, as the proportion of poultry meat consumption has gradually increased, people have paid more and more attention to the development of the poultry industry [1]. In large-scale commercial breeding plants, gastrointestinal diseases are an important factor limiting the development of poultry. In the past few decades, antibiotics have been the most effective measure to solve this problem. Antibiotics can not only effectively inhibit pathogens invasion but also promote the growth of poultry. However, it comes with problems such as various drug resistance, antibiotic residues, and environmental pollution [2]. Because the nontherapeutic use of all antibiotics on animals has been banned in many countries, the development of green, safe, and reliable antibiotic alternatives has become a hot spot. Probiotics are a promising alternative, with the ability to promote growth, inhibit pathogenic microorganisms, and maintain intestinal health [3,4].

With people’s pursuit of food diversity, there has been a rapid increase in the production of duck meat worldwide. The Muscovy duck, as an important economic animal, has a high content of leg and breast muscles and a low content of subcutaneous fat and abdominal fat in the carcass, which meets the meat quality needs of consumers [5]. In addition, it is also rich in various nutrients, such as having a high content of protein and unsaturated fatty acids, various amino acids, iron, zinc, copper, and other minerals, as well as vitamins B and E. [6]. Therefore, Muscovy ducks are popular among consumers.

*Clostridium butyricum* (*C. butyricum*) is a Gram-positive anaerobic bacterium that mainly produces butyric acid with strong tolerance to harsh environments. It is an important probiotic that exists in the intestine of healthy animals and humans [7] and has been widely used in the livestock industry, including in ducks [8]. In previous studies, it has been found that *C. butyricum* has positive effects on weaned piglets [9,10], broilers [11,12], ducks [1], shrimps [13], etc. The positive effects include the promotion of animal growth performance, an increase in feed efficiency, the repair of intestinal barrier functions, the improvement of immunity, the optimization of intestinal microflora structure, and the inhibition of pathogenic bacteria [14,15,16]. The intestinal microbial community of newly born animals is characterized by low diversity and high instability and is susceptible to external factors such as changes in the intestinal environment [17]. Therefore, this period is called a window of opportunity. A recent study showed that *C. butyricum* MIYAIRI 588 (CBM 588) could increase the abundance of *Bifidobacterium*, *Lactobacillus*, and *Lactococcus* in the intestine and also enhance the intestinal barrier function of antibiotic-induced malnourished mice [18]. Another study revealed that *C. butyricum* can effectively reduce the intestinal damage caused by *Salmonella* infection and increase the diversity of intestinal microbes [16]. These indicate that *C. butyricum* could be developed as a strategy of early intervention in the intestinal tract.

As stated above, *C. butyricum* has been commercially developed and widely used in piglets, poultry, and other livestock. Especially for ducks, a 42-day experiment was performed in Peking ducks to illustrate that supplementation with *C. butyricum* up-regulated the average daily weight gain and the activities of antioxidant enzymes [1]. However, studies on the modulation of intestinal microbiota by *C. butyricum* in Muscovy ducks, specifically during early development, are limited. Here, an animal experiment was conducted to assess the effects of the early intervention of *C. butyricum* on shaping the intestinal microbiota of Muscovy ducks, which would provide basic data for the mechanism in *C. butyricum* maintaining gut health.

## 2. Results

### 2.1. Growth Performance

**Figure 1:**
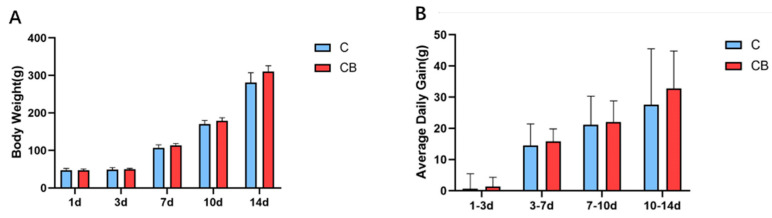
The early intervention of *C. butyricum* had no significant effect on the growth performance of Muscovy ducks. (**A**) The BW of the Muscovy ducks on Days 1, 3, 7, 10, and 14 (displayed as 1d, 3d, 7d, 10d, and 14d, respectively). (**B**) The ADG of the Muscovy ducks in different time periods. Data were expressed as the mean ± SD (*n* = 8) and analyzed by the unpaired two-tailed Student’s *t*-test. C, control group; CB, *C. butyricum* group.

To study whether the early intervention of *C. butyricum* would change the growth performance of Muscovy ducks, we weighed the Muscovy ducks individually on Days 1, 3, 7, 10, and 14. Compared with the C group, the BW of the CB group increased by 2%, 6%, 5%, and 11% at the ages of 3, 7, 10, and 14 days, respectively. However, there was no significant difference (*p* > 0.05; Figure 1A). Similarly, the ADG in the CB group at each time point was higher than in the C group without significant difference (*p* > 0.05, Figure 1B).

### 2.2. Diversity and Structure of the Cecum Microbiota

**Figure 2:**
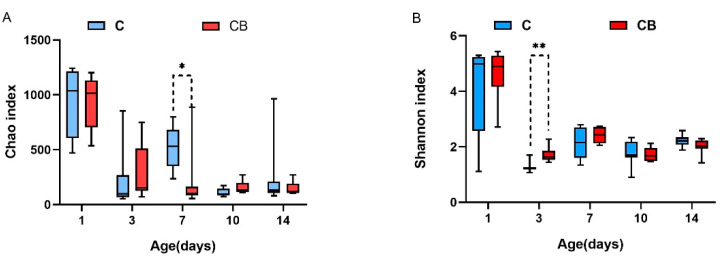
Alpha diversity in the cecal bacterial community of Muscovy ducks in the C and CB groups at the indicated ages. (**A**) The Chao index indicates the species richness of the cecal bacteria. (**B**) The Shannon index shows the community diversity of the cecal bacteria. Asterisks indicate statistically significant differences between the two groups. Data were expressed as the mean ± SD (*n* = 8). Differences between the C and CB groups at each time point were analyzed by the unpaired two-tailed Student’s *t*-test. * *p* < 0.05, ** *p* < 0.01. C, control group; CB, *C. butyricum* group.

To research whether the early intervention of *C. butyricum* would change the diversity and structure of the cecum microbiota in Muscovy ducks, we collected the cecal contents from Muscovy ducks at 1, 3, 7, 10, and 14 days old followed by DNA isolation and 16S rRNA gene sequencing. As shown in Figure 2, early intervention with *C. butyricum* significantly decreased the richness of cecal microbiota on Day 7 (*p* < 0.05) and significantly increased the Shannon index in the CB group compared with that in the C group on Day 3 (*p* < 0.01; Figure 2).

**Figure 3:**
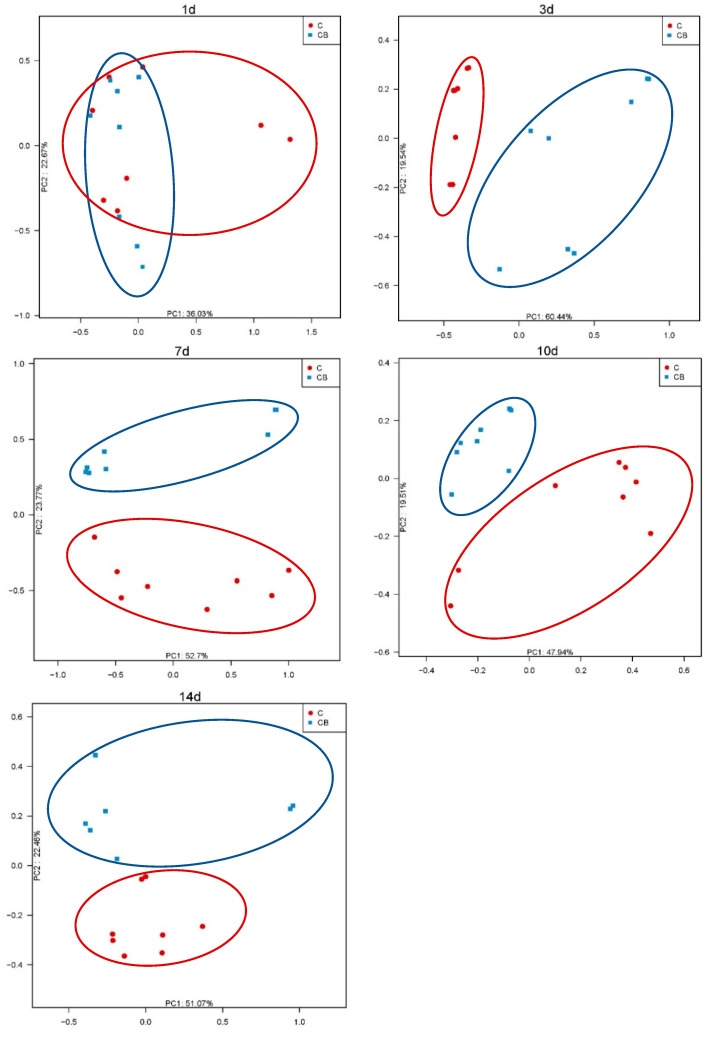
PCoA analysis of β diversity in the cecum bacterial communities of Muscovy ducks in the C and CB groups at the indicated ages. C, control group; CB, *C. butyricum* group. 1d, 3d, 7d, 10d, and 14d represent 1st day, 3rd day, 7th day, 10th day, and 14th day of the present experiment, respectively.

Additionally, to analyze the similarities in microbial communities between the C and CB groups, we performed PCoA with Bray–Curtis dissimilarity on the β diversity of cecal microbiota. The intestinal microflora on Day 1 was significantly different from the microflora at other time points. The microflora of the C and CB groups did not separate from each other on Day 1. With the increase the age, the intestinal microflora gradually matured and stabilized with obvious separation in the microflora (Figure 3).

### 2.3. Cecum Microbiota Composition

**Figure 4:**
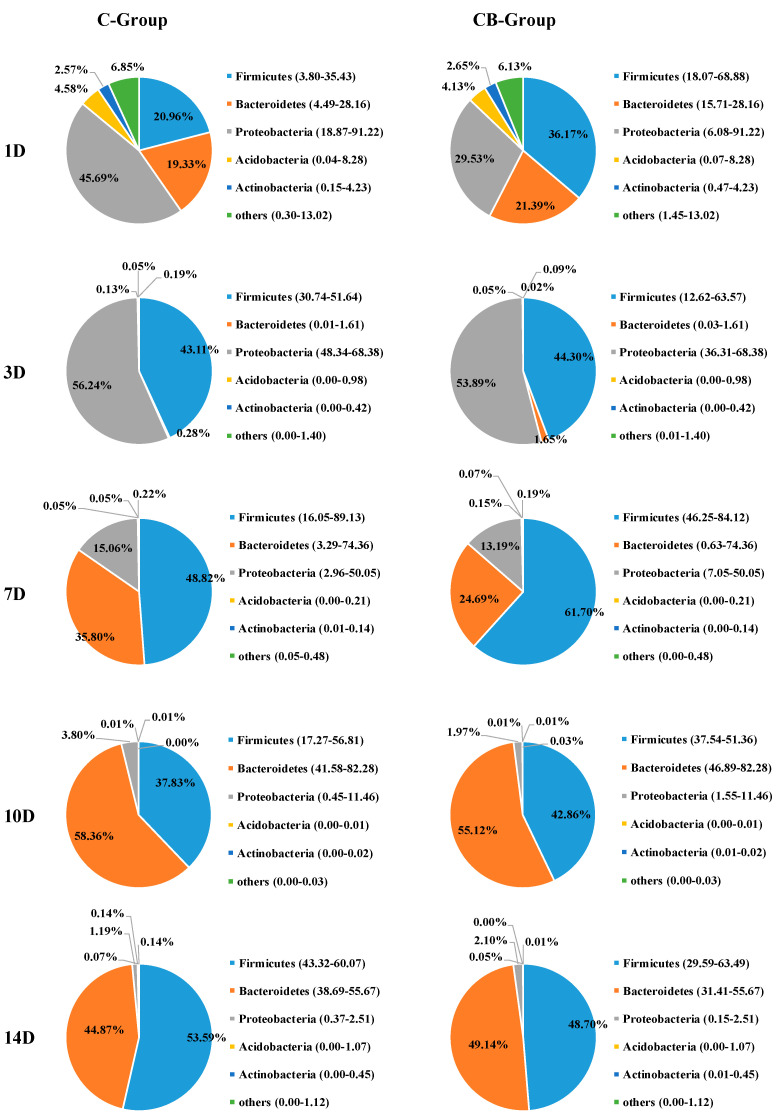
Phylum-level bacterial composition of the cecum microbiota between the C group and the CB group. C group, control group; CB group, *C. butyricum* group. 1D, 3D, 7D, 10D, and 14D represent 1st day, 3rd day, 7th day, 10th day, and 14th day of the present experiment, respectively.

To understand the effect of early intervention with *C. butyricum* on the microbial composition of the cecum, we analyzed the relative abundance of the cecal microbiota at phylum and genus levels at 1, 3, 7, 10, and 14 days old based on the data of 16S rRNA gene sequencing. The cecal microbiota composition of Muscovy ducks was similar at the phylum level between the C and CB groups, but their abundance was very different. The five most abundant phyla across the two groups were *Firmicutes*, *Bacteroidetes*, *Proteobacteria*, *Actinobacteria*, and *Acidobacteria* (Figure 4). From Days 1 to 3, the structure of the microbiota underwent a dramatic alteration. Before Day 3, *Proteobacteria*, *Bacteroidetes*, and *Firmicutes* were the dominant microbiota. However, on Day 3, *Bacteroidetes* decreased drastically, while *Proteobacteria* and *Firmicutes* were the main components of intestinal microbiota. The change gradually stabilized after Day 7. This was also consistent with the results of the β diversity of the cecal microbiota. The inspection of the predicted taxonomic profiles at the phylum level for samples from Days 10 to 14 revealed that the phylum *Bacteroidetes*, with the relative abundance ranging from 49.14% to 55.12%, was the most abundant phylum in the cecal microbiota community of the intervened ducks. *Firmicutes* was the second dominant phylum, with the relative abundance ranging from 42.86% to 58.70%. In contrast, we found *Firmicutes* was the first dominant phylum on Day 7 (61.70%), followed by *Bacteroidetes* (24.69%). During the whole experiment, compared with the CB group, the proportion of phylum *Proteobacteria* observed in the C group was higher. What is more, the abundance of *Proteobacteria* displayed a sustained downward trend with age both in the C and CB groups.

**Figure 5:**
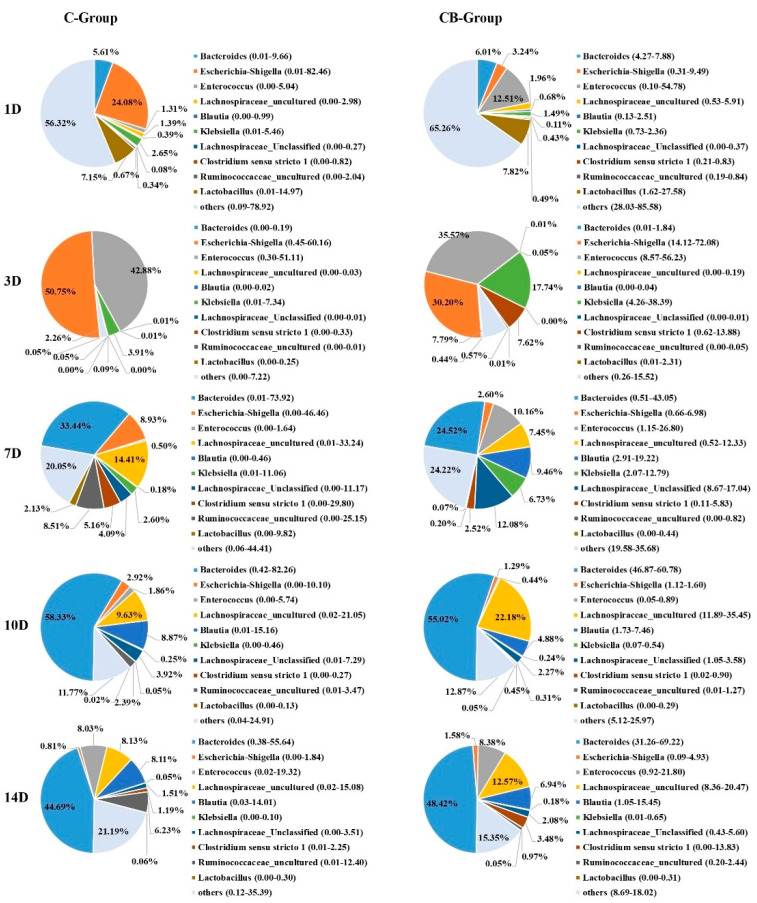
Genus-level bacterial composition of the cecum microbiota between the C group and the CB group. C group, control group; CB group, *C. butyricum* group. 1D, 3D, 7D, 10D, and 14D represent 1st day, 3rd day, 7th day, 10th day, and 14th day of the present experiment, respectively.

At the genus level, the bacterial taxa were quite different at all time points between the C group and the CB group. However, their changing trend was the same as that of the phylum level. The relative abundances of the most abundant genera were *Bacteroides*, *Escherichia–Shigella*, *Enterococcus*, *Lachnospiraceae*_uncultured, *Klebsiella*, *Lachnospiraceae*_Unclassified, and so on (Figure 5). *Bacteroides* became the most abundant genus on Day 7, and its abundance increased with age reaching the peak on Day 10. *Bacteroides* in Muscovy ducks of the C group and the CB group accounted for 58.33% and 55.02%, respectively. The abundance of *Klebsiella* showed an increasing trend from Days 1 to 3 and then decreased from Days 7 to 14. *Escherichia–Shigella* was the most predominant genus in the cecum of Muscovy ducks in the C group but the second most abundant genus in the CB group on Day 3 with the level decreasing with increasing age. More importantly, from Days 3 to 10, the abundance of *Escherichia–Shigella* in the CB group was always lower than in the C group. In contrast, *Lachnospiraceae*_uncultured had a lower relative abundance in the early stage of the study but were predominant from Days 7 to 14 in both the C and CB groups.

**Figure 6:**
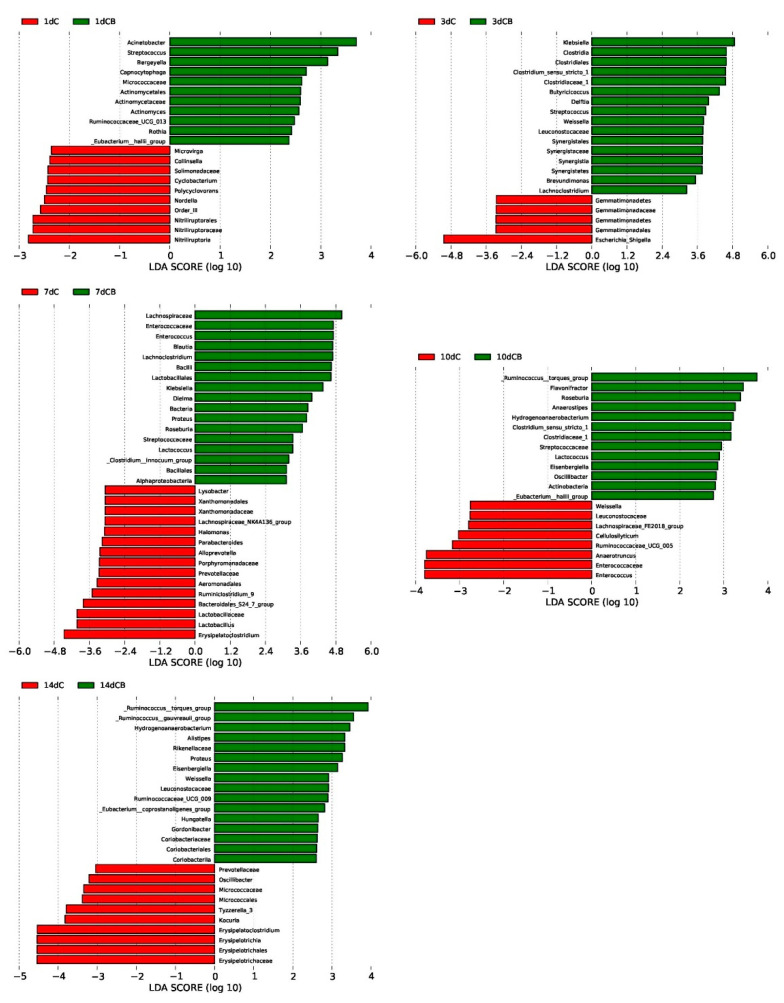
Differentially abundant bacteria between the C group and the CB group at each sampling time point. Histograms of linear discriminant analysis (LDA) scores (threshold = ≥2) on Days 1, 3, 7, 10, and 14 (displayed as 1d, 3d, 7d, 10d, and 14d, respectively) are plotted. C, control group; CB, *C. butyricum* group.

LefSe analysis was used to further determine the significant difference in the relative abundance of bacteria in the cecum microflora of the C group and the CB group at a certain age, which highlighted the statistical significance and further proving that the intestinal microflora was biologically consistent. Based on the logarithmic LDA score of 2.0 as the cutoff, we found that 21 taxa on Days 1 and 3 were significantly affected by early intervention, followed by 32 taxa on Day 7, 21 taxa on Day 10, and 26 taxa on Day 14 (Figure 6). Similar to the results of cecal microbiota composition, the LDA score of *Klebsiella* was the highest on Day 3 but decreased on Day 7 with the absence on the list of significantly different genera on Days 10 and 14. Nevertheless, the LDA score of the genus *Escherichia–Shigella* was significantly higher in the ducks of the C group than in the CB group on Day 3. The *Ruminococus*_torques_group showed up in the list of most different genera in the CB group on Day 10 and kept as the top one in the CB group.

### 2.4. Core Microbial Genera in the Cecum Contents of the Ducks

**Figure 7:**
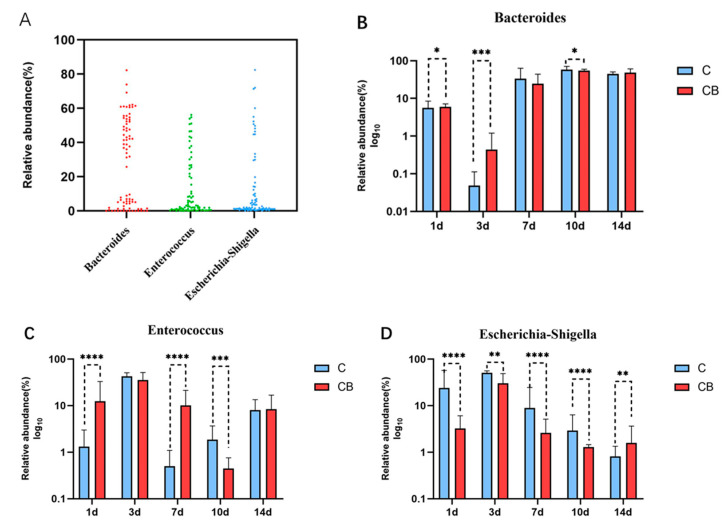
Core microbial taxa in the cecum of Muscovy duck. (**A**) Core microbial genera in the cecum of the Muscovy ducks. (*n* = 80). (**B**–**D**) Relative abundance of each core genus in the C and CB groups at each sampling time point. Data were expressed as the mean ± SD (*n* = 8) and analyzed by the unpaired two-tailed Student’s *t*-test. * *p* < 0.05, ** *p* < 0.01, *** *p* < 0.001, **** *p* < 0.0001. C, control group; CB, *C. butyricum* group. 1d, 3d, 7d, 10d, and 14d represent 1st day, 3rd day, 7th day, 10th day and 14th day of the present experiment, respectively.

To identify the core microflora in the cecum of Muscovy ducks, we screened out the 3 genera shared among all of 80 cecal content samples (8 replications per group on Days 1, 3, 7, 10, and 14), which could be considered as the basic genera for studying the cecal microbiota of Muscovy ducks. The result showed that three dominant genera were found in all sample individuals (*n* = 80), namely *Bacteroides*, *Enterococcus*, and *Escherichia–Shigella* (Figure 7A). These three core genera belong to three phyla. *Enterococcus* genus was from the phylum *Firmicutes*, while *Bacteroides* and *Escherichia–Shigella* were from the phylum *Bacteroidetes* and the phyla *Proteobacteria*, respectively. The relative abundance of these genera changed greatly with age with consistent detection in the cecum contents of ducks from Days 1 to 14, which indicated that early intervention with *C. butyricum* changed the relative abundance but did not change the existence of these specific microbial genera. The relative abundances of the three core microbial genera across the two groups at the indicated ages are shown in Figure 7B–D. Generally speaking, the relative abundance of *Bacteroides* was reduced on Day 3, increased back on Day 7, and stabilized until the end of the experiment, while the relative abundance of *Enterococcus* and *Escherichia–Shigella* shared a trend that reached a peak on Day 3 and gradually decreased until Day 14 (Figure 7B–D). Furthermore, the relative abundance of *Bacteroides* in the CB group was significantly higher on Days 1 (*p* < 0.05) and 3 (*p* < 0.001) but was lower on Day 10 (*p* < 0.05) than in the C group (Figure 7B). For *Enterococcus* compared with the C group, the relative abundance of *Enterococcus* in the CB group was significantly higher on Days 1 and 7 (*p* < 0.001) but was lower on Day 10 (*p* < 0.001). Interestingly, throughout the experiment, the relative abundance of *Escherichia–Shigella* was always significantly different between the two groups. At the beginning (from Days 1 to 10), the abundance of *Escherichia–Shigella* in the C group was constantly higher than in the CB group with an opposite result at the end of the experiment (Day 14).

### 2.5. Short-Chain Fatty Acid Levels in Cecum

**Figure 8:**
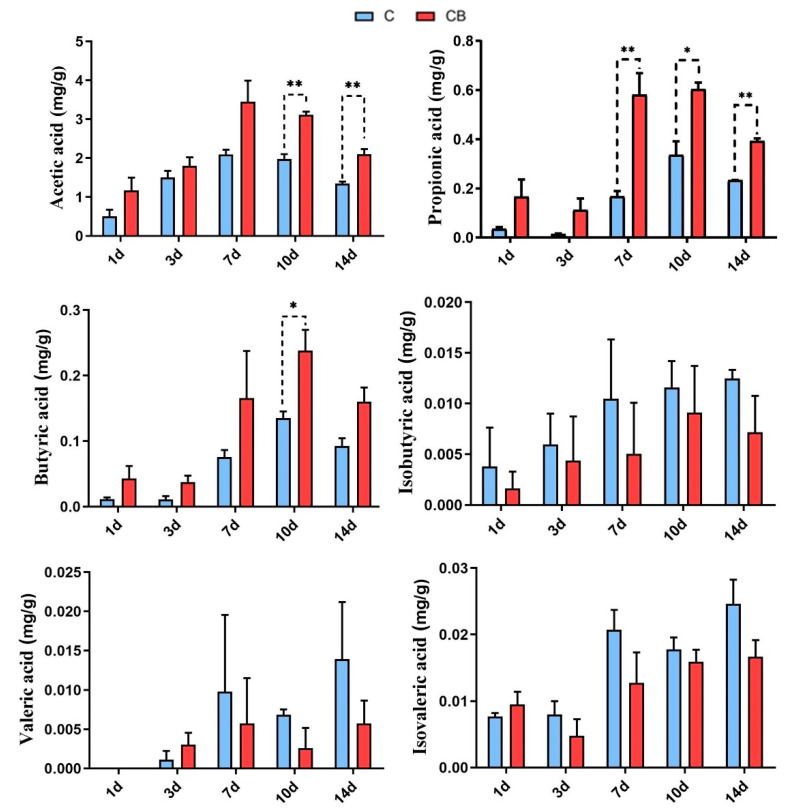
The concentrations of short-chain fatty acids (SCFAs) in the cecum of the Muscovy ducks in the C group and the CB group. Data were expressed as the mean ± SD (*n* = 8). Differences between the C and CB groups at each time point were analyzed by the unpaired two-tailed Student’s *t*-test. * *p* < 0.05, ** *p* < 0.01. C group, control group; CB group, *C. butyricum* group. 1d, 3d, 7d, 10d, and 14d represent 1st day, 3rd day, 7th day, 10th day, and 14th day of the present experiment, respectively.

To examine the impact of early intervention with *C. butyricum* on short-chain fatty acids (SCFAs) in the cecum of Muscovy ducks, we determined the SCFA contents in the cecal content samples by the gas chromatography analysis. The concentrations of acetic, propionic, and butyric acids in the cecum of the *C. butyricum*-treated ducks were increased compared with those in the C group. Among them, the concentration of acetic acid in the CB group was significantly higher (*p* < 0.01) than in the C group on Days 10 and 14. Moreover, the content of butyric acid in the CB group was significantly higher (*p* < 0.05) than in the C group on Day 10. However, the concentrations of isobutyric, valeric, and isovaleric acids presented no significant change (*p* > 0.05) between the C group and the CB group (Figure 8).

## 3. Discussion

Muscovy duck, since it was introduced to China, has become one of the main meat sources in the market due to its good meat quality, high nutritional value, and delicious taste [19]. However, the production of Muscovy ducks has been severely affected by gastrointestinal diseases. *C. butyricum* has been one of the popular strategies to maintain the gut health of poultry in recent years [11,20,21]. This investigation aimed to determine whether early intervention with *C. butyricum* could have a beneficial effect on the development of intestinal microbiota in Muscovy ducks. We found that early intervention with *C. butyricum* modulated the intestinal microbiota structure of Muscovy ducklings without significant effects on growth performance. However, several studies have proved that *C. butyricum* could promote growth performance [1,9,10,11,12,13]. This might be due to the different supplementary ways and duration of *C. butyricum* administration.

In the α diversity analysis, the highest levels of richness and diversity of the cecum microbiota in the ducks were found on Day 1, followed by a clear downward trend after that. We speculate that this result may be caused by the bacteria on the eggshell [22]. Before the Muscovy ducks hatched, some of the bacteria remaining on the eggshells could survive in the form of spores and could be ingested by newborn animals during hatching. In addition, our results showed that the Shannon index was significantly lower in the C group on Day 3 compared to the CB group (Figure 2). One of the possible reasons might be the administration of *C. butyricum*. As we all know, butyric acid, a product of *C. butyricum*, can lower the pH of the intestinal tract, thereby inhibiting the growth of pathogens [7]. Moreover, we observed that the species richness of gut microbiota was significantly higher in the C group than in the CB group on Day 7 (Figure 2). We speculate that the colonization of *C. butyricum* might compete with other bacteria and decrease the microbiota diversity. Therefore, the low species richness of the CB group may be attributed to the enrichment of *C. butyricum*. Interestingly, it was found in some previous studies that α diversity generally increases with age [23], which was believed to contribute to maintaining the growth and health of animals [24,25]. In contrast, a recent study indicated that inoculation with cecal fermentation broth lowered the α diversity of cecal microbiota, consistent with our results. Because some microbes are almost useless to the host, sometimes a relatively low α diversity is desirable [26]. The β diversity between the two groups at all sampling time points was compared using PCoA of the unweighted UniFrac distance. These PCoA plots showed that the microbial communities of the two groups had not yet been separated on Day 1 (Figure 3). Nevertheless, starting from Day 3, the two groups of microorganisms clearly separated (Figure 3). This indicated that the early intervention of *C. butyricum* was effective.

The gastrointestinal tract of poultry is closely linked to the health of the host, especially the composition of the cecum microorganisms [27]. In this study, the dominant phyla detected in the cecum contents were the phyla *Bacteroidetes*, *Firmicutes*, and *Proteobacteria* (Figure 4), which is consistent with previous studies [28,29]. Firmicutes becoming the dominant phylum in the intestines of poultry may be related to the anaerobic environment formed during intestinal development [30]. Moreover, the phylum *Firmicutes* has been associated with fiber digestion and short-chain fatty acid metabolism, especially for the synthesis of butyrate [9]. Similarly, the *Bacteroides* phylum could degrade indigestible carbohydrates and played an important role in immune regulation and improvement of the intestinal mucosa [31]. We further found that early intervention of *C. butyricum* could change the relative abundances of dominant phyla, as shown by the proportion of *Proteobacteria* in the CB group, which was always lower than the C group. The decrease in the relative abundance of the phylum *Proteobacteria* may indicate that the ducks after intervention had a healthier intestinal environment because of the wide variety of pathogenic bacteria in this phylum [32].

At the genus level, the most valuable result was the discovery that the proportion of *Escherichia–Shigella* was significantly reduced after early intervention, especially on Day 1 (Figure 5). The genus *Escherichia–Shigella*, as one of the most common pathogens that cause poultry diseases, exists widely in the environment. Previous studies have documented that virulence factors produced by the genus *Escherichia–Shigella* can break the intestinal mucosal barrier, cause diarrhea, affect immune function, and cause inflammation [33]. Not surprisingly, the relative abundance of *Escherichia–Shigella* in the cecum contents of ducks was found to be negatively correlated with age (Figure 7). From Day 7, the proportion of *Escherichia–Shigella* in the two groups was significantly reduced (Figure 5). As for the sudden increase in *Escherichia–Shigella* on the third day, we suspect that it may be due to the immatureness of the immune barrier function of ducklings when the oral gavage was performed. These results indicate the sensitivity of the intestinal microflora of Muscovy ducks during early development. In addition, another difference in the composition of the cecum microbiota detected at the genus level was *Bacteroides*. Throughout the experiment, *Bacteroides* was the main genus. The benefits of *Bacteroides* are well known. They can effectively degrade long-chain polysaccharides as well as actively improve the intestinal environment for beneficial microorganisms [31].

Additionally, LefSe analysis identified other representative species as biomarkers to distinguish the microbiota of the two groups. For example, *Klebsiella* and *Enterococcus*, as members of the *Enterobacteriaceae* family, are pathogenic bacteria in the intestinal tract. Although they appeared in the early stages of ducks after the intervention, they gradually decreased with the appearance of lactic acid bacteria (*Lactobacillus*, *Lactococcus*). Previous research showed that lactic acid bacteria may affect the colonization pattern of *Enterococcus* and increase the nutrient absorption of energy and minerals [34]. Additionally, the family *Ruminococcaceae*, as a member of SCFA production [35], was significantly enriched in the intervened ducks, and these bacteria are considered to be dominant players in the degradation of diverse polysaccharides and fibers [36]. As the study progressed, we found that the relative abundance of *Ruminococcaceae* was still higher than the C group. In summary, the results of screening representative species show that early intervention effectively promotes intestinal health by promoting the growth of beneficial species and inhibiting the proliferation of pathogenic bacteria.

As for the core microbiome, three genera were detected in all of the ducks, regardless of group or age, and were identified as the core cecum microbiomes in ducks. The result was that they came from the phylum *Firmicutes*, *Bacteroides*, and *Proteobacteria*, which was consistent with the results of the dominant bacteria phyla. However, these core genera also included some pathogenic bacteria. However, it could be seen from Figure 4 that they always only occupy a small proportion, suggesting that the pathogenicity may be related to the abundance of pathogenic bacteria. Therefore, this may imply that early intervention can be used to reduce the abundance of colonized pathogens and improve the intestinal health of ducks.

The SCFAs, in particular acetate, propionate, and butyrate, the main end product metabolized by microorganisms in the large intestine, are an important energy source for intestinal cells and are vital to the health of the host [37]. In this study, we analyzed the six main species of SCFAs in cecum. The results showed that the SCFAs of the ducks were increased by *C. butyricum* administration, which was consistent with studies of *C. butyricum* in other animals [9,10,11]. This may be due to the early intervention of *C. butyricum* altered the structure of intestinal microbes.

In this study, we found that propionic acid and *Bacteroides* increased significantly by *C. butyricum* treatment at the same time point. The *Bacteroides* phylum is recognized as propionic-acid-producing bacteria phylum. Furthermore, *C. butyricum* may also have a regulatory effect on the intestinal microflora of the experimental ducks. The *C. butyricum*, as a probiotic that produces butyric acid, has been developed to regulate the intestinal microflora in the livestock industry. For instance, in chickens with necrotizing enteritis model, *C. butyricum* reduced the abundance of C. perfringens in the intestine [21]. Another study showed that the combined use of *C. butyricum* and *Enterococcus faecalis* increased the abundance of *Firmicutes* and *Proteobacteria* in the intestine [9]. In addition, an increased SCFA concentration can increase intestinal acidity, promote the secretion of anti-inflammatory factors, and increase the ratio of villi height to crypts in the intestine, which are related to antibacterial properties, immunity, and digestion and absorption, respectively [37]. Therefore, we suggest that the early intervention of *C. butyricum* can increase the SCFAs in the cecum content of ducks and protect the health of the intestines.

## 4. Materials and Methods

### 4.1. Animal Experimental Design

All procedures of the animal experiment in this study were approved by the animal welfare committee of the Zhejiang Academy of Agricultural Sciences. A total of 160 male 1-day-old Muscovy ducks with an initial body weight (BW) of 47.3 ± 3.9 g were obtained from Hewang Poultry Industry Co., Ltd. (Lanxi County, Jinhua City, Zhejiang Province, China) and were randomly divided into two groups: the control group (C group) and the *C. butyricum* group (CB group). Each group had 8 replications (cages), 10 ducks per replication (cage). For the CB group, 1 mL of *C. butyricum* solution (≥2 × 10^9^ CFU/mL) was administered individually within 2 h immediately after hatching. *C. butyricum* was cultured from *C. butyricum* spore tablets (*C. butyricum* ≥ 0.35 × 10^6^ CFU per tablet, Miyarisan Pharmaceutical, Tokyo, Japan). A volume of 1 mL of normal saline was given to the ducks of the C group in the same manner. Ducks were administered once a day, and the administration lasted for 3 days. The ducks were raised in cages with free access to commercial feed (Table 1) and drinking water under a standard commercial condition in the Laboratory Animal Center of Zhejiang Academy of Agricultural Sciences (ZAASDLSY2019-3640).

### 4.2. Sample Collection

All ducks were weighed individually, and the ADG for each duck was calculated on Days 1, 3, 7, 10, and 14. Eight ducks were randomly selected from each group (1 per cage) at the ages of 1, 3, 7, 10, and 14 days for sample collection and were euthanized by CO_2_ asphyxiation. After being weighed and slaughtered, the cecum segments and the contents of the cecum were collected, immediately frozen in liquid nitrogen, and then transferred to a −80 °C freezer until RNA or DNA isolation.

### 4.3. DNA Extraction and Purification

ZR Fecal DNA MiniPrep Kit (Zymo Research, Tustin, CA, USA) was used to extract the cecal microbial genomic DNA from each cecal content of Muscovy ducks. The V4-V5 region of 16S rRNA was PCR amplified using primers 515F (5′-GTGCCAGCMGCCGCGGTAA-3′) and 806R (5′-GGACTACHVGGGTWTCTAAT-3′). The reaction conditions were as follows: 95 °C for 3 min; 25 cycles at 95 °C for 30 s, 55 °C for 30 s, and 72 °C for 45 s; and 72 °C for 10 min. The PCR products were purified using the AxyPrep DNA Gel Extraction Kit (AXYGEN, Union City, CA, USA) and then quantified using QuantiFluor-ST (Promega, Madison, WI, USA).

### 4.4. 16S rRNA Gene Sequencing and Data Processing

An Illumina TruSeq DNA PCR-Free Library Preparation Kit (Illumina) was applied for sequencing library generation. The quality of the generated library was evaluated by using a Qubit 2.0 Fluorometer (Thermo Scientific, Waltham, MA, USA) and an Agilent Bioanalyzer 2100 system. The qualified library was sequenced commercially by Mingke Biotechnology (Hangzhou, China) on an Illumina HiSeq platform, generating 250 bp paired-end reads. The Illumina paired-end reads were filtered in Quantitative Insights into Microbial Ecology (QIIME) quality filters to remove low-quality reads [38] and then merged into a sequence with a minimum overlap length of 10 bp using FLASH [39]. The UPARSE and UCHIME were used for read clustering and the cutoff (based on 97% similar identity) for operational taxonomic units (OTUs) and removing chimeric OTUs. As per the results of OTU clustering, corresponding species information and species-based abundance distributions can be obtained by using species annotations created for each OTU sequence. Alpha diversity (Chao1 and Shannon indexes), LefSe analysis, and principal coordinates analysis (PCoA) were performed in the R project. The raw 16S rRNA gene sequencing data are available in the SRA database under Accession Number PRJNA738619.

### 4.5. Short-Chain Fatty Acids Analysis

SCFAs concentration, including acetic, propionic, isobutyric, butyric, isovaleric, and valeric acids, in the cecum were assayed by gas chromatography as previously described [26]. Briefly, 0.10 g of cecal content was added into a 1.5 mL sterile centrifuge tube. A 1 mL volume of sterile PBS solution was added into the tube followed by vortexing until it was uniform. The mixture was then centrifuged at 7000× *g* rpm for 10 min, and 500 μL of the supernatant was added into a new 1.5 mL centrifuge tube with the addition of 100 μL of 25% metaphosphate crotonic acid solution. After being mixed well, the mixture was stored at −20 °C for 24 h. After being thawed and centrifuged at 14,000× *g* rpm for 2 min, the supernatant was filtered through a 0.22 μm membrane and injected into a Shimadzu GC-2010 ATF instrument for gas chromatographic analysis. The carrier gas was H_2_, the temperature of the injector was 170 °C, and the detector temperature was 250 °C. The heating program: the started temperature was 70 °C, heated to 180 °C at a rate of 15 °C min^−1^, kept at 180 °C for 3 min, then increased to 250 °C at 40 °C min^−1^ for 5 min.

### 4.6. Statistical Analyses

Effects of *C. butyricum* on the cecal microbiota were examined using PCoA, which was computed based on Bray–Curtis dissimilarities. The data, including growth performance, the relative abundance of bacteria, and the concentrations of SCFAs, were analyzed by the unpaired two-tailed Student’s *t*-test with SPSS 19.0 software (IBM, New York, NY, USA) and were expressed as the mean ± standard deviation (SD). *p* < 0.05 indicated a significant difference, while *p* < 0.01 indicated an extremely significant difference. GraphPad Prism 6 (GraphPad software, San Diego, CA, USA) was used for graph generation.

## 5. Conclusions

In summary, early intervention with *C. butyricum* in Muscovy ducks can regulate the structure and composition of the gut microbiota. The decrease in pathogenic bacteria, the increase in beneficial bacteria, and the increase in the concentration of SCFAs are beneficial to duck intestinal health. Therefore, it may be possible to develop new intervention strategies to induce desirable changes in the gut microbiota to enhance the growth and productivity of poultry, as well as other animals and humans.

## Figures and Tables

**Table 1:** Diet composition and nutritional composition.

Items	Contents
**Ingredients (air-dried basis, %)**	
Corn	54.50
Soybean meal	21.00
Wheat middlings	10.00
DDGS	4.50
Fish meal	3.00
Rapeseed meal	3.00
Soybean oil	1.50
Dicalcium phosphate	0.75
Methionine	0.23
Lysine	0.32
Salt	0.20
Premix ^1^	1.00
**Nutrient Content ^2^**	
Crude protein, %	19.50
Metabolizable energy, MJ/kg	12.12
Lysine, %	0.95
Methionine + cysteine, %	0.68
Ca, %	0.86
Total phosphorus, %	0.40

^1^ Concentrate mixture provided the following per kilogram of complete diet: vitamin A, 9000 IU; vitamin D3, 6000 IU; vitamin E, 30 mg; vitamin K3, 2.0 mg; vitamin B1, 8.0 mg; vitamin B2, 4.0 mg; vitamin B6, 8.0 mg; vitamin B12, 0.9 mg; choline, 500 mg; niacin, 45 mg; pantothenic acid, 15 mg; folic acid, 0.8 mg; biotin, 0.3 mg; iron, 70 mg; copper, 7.5 mg; manganese, 80 mg; zinc, 65 mg; iodine, 0.35 mg; selenium, 0.25 mg. ^2^ Nutritional ingredients are calculated values.

## Data Availability

The raw 16S rRNA gene sequencing data are available in the SRA database under Accession Number PRJNA738619.
